# Biomedical and Textile Applications of *Alternanthera sessilis* Leaf Extract Mediated Synthesis of Colloidal Silver Nanoparticle

**DOI:** 10.3390/nano12162759

**Published:** 2022-08-12

**Authors:** Nivedhitha Kabeerdass, Karthikeyan Murugesan, Natarajan Arumugam, Abdulrahman I. Almansour, Raju Suresh Kumar, Sinouvassane Djearamane, Ashok Kumar Kumaravel, Palanivel Velmurugan, Vinayagam Mohanavel, Subbiah Suresh Kumar, Selvaraj Vijayanand, Parasuraman Padmanabhan, Balázs Gulyás, Maghimaa Mathanmohun

**Affiliations:** 1 Department of Microbiology, Muthayammal College of Arts and Science, Rasipuram, Namakkal 637 408, Tamilnadu, India; 2 Department of Microbiology, Faculty of Medicine, Quest International University, Ipoh 30250, Malaysia; 3 Department of Chemistry, College of Science, King Saud University, P.O. Box 2455, Riyadh 11451, Saudi Arabia; 4 Department of Biomedical Science, Faculty of Science, Universiti Tunku Abdul Rahman, Kampar 31900, Malaysia; 5 Systems Biology Lab, School of Chemical Engineering & Bio-Engineering, University of Ulsan, Daehak-ro, Nam-gu, Ulsan 680-749, South Korea; 6 Centre for Materials Engineering and Regenerative Medicine, Bharath Institute of Higher Education and Research, Chennai 600073, Tamilnadu, India; 7 Department of Biotechnology, Thiruvalluvar University, Serkkadu, Vellore 632115, Tamilnadu, India; 8 Lee Kong Chian School of Medicine, Nanyang Technological University, Singapore 636921, Singapore; 9 Cognitive Neuroimaging Centre, Nanyang Technological University, 59 Nanyang Drive, Singapore 636921, Singapore; 10 Department of Clinical Neuroscience, Center for Psychiatry Research, Karolinska Institute and Stockholm County Council, SE-171 76 Stockholm, Sweden

**Keywords:** plant extract, antibacterial, SEM with EDAX, HR-TEM, fabrics

## Abstract

The aqueous extract of *Alternanthera sessilis* (As) acts as the precursors for the quick reduction of silver ions, which leads to the formation of silver nanoparticles. In the agar, well diffusion method of the *Klebsiella pneumoniae* shows the minimal inhibitory concentration of 12 mm against *A. sessilis* mediated silver nanoparticles (As-AgNPs) at 60 µg/mL concentration. Fabric treated with novel AS-AgNPs is tested against the *K. pneumoniae* and shows an inhibitory action of 12 mm with mixed cotton that determines the antimicrobial efficacy of the fabrics. Uv- visible spectrophotometer was performed, showing a surface plasmon resonance peak at 450 nm cm^−1^. FTIR shows the vibration and the infrared radiation at a specific wavelength of 500–4000 cm^−1^. The HR-TEM analysis showed the presence of black-white crystalline, spherical-shaped As-AgNPs embedded on the fabrics range of 15 nm–40 nm. In the scanning electron microscope, the presence of small ball-shaped As-AgNPs embedded on the fabrics at a voltage of 30 KV was found with a magnification of 578X. EDAX was performed in which the nanoparticles show a peak of 2.6–3.9 KeV, and it also reveals the presence of the composition, distribution, and elemental mapping of the nanoparticles. The cytotoxic activity of synthesized nanosilver was carried out against L929 cell lines, which show cell viability at a concentration of 2.5 µg mL^−1^. Cell proliferation assay shows no cytotoxicity against L929 cell lines for 24 h. In this study, the green synthesis of silver nanoparticles from *A. sessilis* appears to be a cheap, eco-friendly, and alternative approach for curing infectious ulcers on the floor of the stratum corneum. Nanotechnology conjoined with herbal therapeutics provides a promising solution for wound management.

## 1. Introduction

The stratum corneum is the primitive barrier in the human body that protects against environmental factors and dermal pathologies. Increasing the penetration and retention of the active ingredient through the skin barrier is the biggest challenge of the stratum corneum. Restoring the integrity of the skin and subcutaneous tissue and maintaining the complex sequence of the well-assembled biochemical and cellular phenomenon is called the wound healing process. Sparse sterilization leads to accumulation of infectious microbes’ cause pathogenicity on the interrupted pellicle, which occurred incidentally or accidentally. New products are obtained at the nano level in the fast-growing field of nanotechnology. The metal nanoparticles show much importance to catalytic, magnetic, and optical properties. Among many noble metal nanoparticles, silver is the most efficient and possesses excellent antimicrobial, anti-inflammatory, and antioxidant properties. Silver ions are known (1–100 nm) for centuries for their antimicrobial property. Silver ions enter the cell, pass the cell membrane barrier, bind to the ions, and denature the proteins, which is the key to cellular activity. Through microemulsion, UV irritated, and photoinduced reduction are the chemical methods applied for the synthesis of silver nanoparticles. Polymeric compounds act as protective agents to stabilize silver nanoparticles. However, in the case of medicinal properties, rich plant-mediated silver nanoparticles with flavonoids, sugars, and anthraquinone are used to reduce the metal ions. Conductivity, chemical stability, and antimicrobial activity are exposed by silver in the colloidal state. Recent work reports that the capability of the metal nanoparticle is synthesized using single-celled organisms and using plant extracts like *Catharanthus* *roseus* [1], *Curcuma longa* [2], *Jatropha curcus* [3], Aloe vera [4], and *Alternanthera sessilis* (Linn.) [5].

The present work focuses on the medicinal properties of *A. sessilis* extract mediated AgNPs, which is a better therapeutic agent for repairing the sore. The Phytochemicals present in the plant extracts act as the dropping and capping agents. A perennial herb, with simple green leaves, white flowers, and bearing the short petiole is known as *A. sessilis* found all over India. Anti-inflammatory, antioxidant, and wound repairing efficacy; all these properties are categorized in the herb *A. sessilis*. The tocopherol-like [6] antioxidant in *A. sessilis* inhibits the oxidative chain reaction catalyzed by the free radicals for the biomolecule’s destruction. Vanillic acid, ethylgallate, apigenin, chlorogenic acid, catechin, and epigallocatechin are some of the bioactive constituents in the *A. sessilis* showing anti-inflammatory activity [7] and also help in repairing the tissues [8,9]. The other medicinal properties exposed in the leaves are used to treat eye diseases, the antidote for poisonous reptile bites, treat indigestion, and many chronic skin diseases. Different chemical components present in the *A. sessilis* herb are proteins, ascorbic acid, sterols, phenols, alkaloids, ascorbic acid, fatty acids, and carotenes; all these constituents act as reducing agents for the synthesis of AgNPs. Unique and unusual physicochemical properties of metallic nanoparticles bearing one dimension of 1–100 nm show attention to both scientific and technological fields [10]. The specific size, shape, and circulation of nanoparticles made it a novel and promising agent in medical application. Antibacterial textile surfaces can be created by incorporating plants mediated silver nanoparticles agent into the fabric. With known antibacterial activity, they are accomplished by blending agents like a sliver into the polymer. Leaching out these agents from the fibrous matrix at a slow rate kill or inhibits the microbes in the local environment. Skin cancer is a dreadful disease that leads to death among millions of people. L 929 cell lines are procured from the subcutaneous connective tissue of the mouse. It is a normal fibroblast cell line and has the characteristic feature of cancer. Fibroblast acts in framing the structure of tissues in animals. It plays a key role in the repair of tissue [11]. 

In this study, the green synthesized mediated nanoparticles are treated against the L 929 cell line for cell toxicity. Unique characteristics of the silver nanoparticles act as the therapeutic barrier for wound healing to reach its target site [12]. 

## 2. Materials and Methods

### 2.1. Collection of Pathogenic Strains

Gram-positive and Gram-negative bacterial samples which are collected in sterile tubes, and unbreakable screw-capped bottles are isolated, subcultured, and maintained in the icebox of the various hospitals and laboratories in Madurai (India) [13]. These samples are shifted to the college laboratory for processing in time.

### 2.2. Isolation and Screening

Isolated the above bacterial samples in their respective media and incubated at 37 °C for 24 h. Then, the colony morphology, gram staining, and biochemical tests are done, to identify the number of isolates, whether it is Gram-positive or Gram-negative, and the pure culture plates are sealed, labeled, and stored in the icebox for further analysis [14,15].

### 2.3. Floral Collections

A cost-effective, viridescent, stalkless, perennial herb (leaf) is plucked in the ponds, and canals from the nearby villages of Kolli Hills, Rasipuram, Namakkal (India) [16]. Then, surface sterilization is done by rinsing in sterile water and separating the affected leaf. The clean, unaffected leaves are then dried in the shade, pulverized, and made into fine crystals for extraction.

### 2.4. AS-AgNPs Synthesis (A. sessilis Mediated Silver Nanoparticles Synthesis)

After finding the dry weight, 50 g of *A. sessilis* powder was treated in 500 mL of water in an Erlenmeyer flask and stirred continuously into a concentrated solution. Then, the beaker was placed in a heating mantle at 90 °C for 30 min and the parameters were optimized [17]. Then, the precursor silver nitrate at a concentration of 1 mM was mixed gently. In the absence of light after 72 h, the color change from straw to chocolate brown was monitored at regular intervals, which indicates the presence of the silver nanoparticles [18].

### 2.5. Antibacterial Activity

In this minimal inhibitory concentration, Gram-negative and Gram-positive bacteria show a zone of inhibition at various concentrations (such as 20 µg, 40 µg, and 60 µg), indicating the susceptibility and resistant action of the pathogens loaded on the wells made by 6 mM Cork borer [19]. 

### 2.6. Embedding Synthesized As-AgNPs to Fabrics

The procured cotton and mixed cotton fabrics were sterilized, soaked in 10 mL of *A. sessilis* mediated silver nanoparticles (As-AgNPs). Lastly, the As-AgNPs embedded fabrics were removed and cleansed in deionized water subsequently dehydrated at 37 °C. Now, the As-AgNPs embedded fabrics are ready to proceed with the antibacterial activity.

#### Fabric Sensitivity Test

In the freshly prepared Muller Hinton agar (MHA) plate, the pathogens are spread evenly by using a sterile dry swab, and then fabrics embedded synthesized As-AgNPs are placed in the plate [20], where non-coated, AS-AgNPs fabrics act as a control. 

### 2.7. Conformational Study of AS-AgNPs Coated on Fabrics

#### 2.7.1. UV-Visible Spectroscopy

The silver nanoparticle was analyzed in UV3600-UV–VIS-NIR spectrophotometer (Systronics sys119, Ahmedabad, India). The reaction mixture was taken in a quartz cuvette for spectral analysis. Spectroscopic readings are taken at different time intervals and concentrations. The spectral analysis was obtained at various wavelengths corresponding to its intensity of absorbance [21].

#### 2.7.2. FTIR

Fourier Transform Infrared Spectroscopy (FTIR) was performed to identify the possible functional groups in biomolecules present in the plant extract. It is used to determine the phyto components present in the sample, many scans are taken, and their resolution and the spectral range are measured [22]. 

#### 2.7.3. SEM with EDAX (Energy Dispersive X-ray Analysis)

SEM (TSCAN (Floor Model, US) was used to visualize the surface morphology of the samples. The image obtained at a high resolution enhance the important information like size, shape, composition, and other properties of the nanoparticles. A beam of electrons was focused on the sample after the emission of the characteristic X rays from the specimen. Metal nanoparticles are measured quantitatively and qualitatively through energy dispersive X-ray spectrometers [23]. 

#### 2.7.4. HR-TEM

A crystallographic structure of the image at an atomic level is recorded at high magnification and resolution with the help of a beam of electrons [24]. The sample is made into thin sections using diamond abrasives, mounted, and finally the image in nanometer-scale against the black and white background is observed.

#### 2.7.5. Cell Viability Assay

MTT cell assay (colorimetric assay for assessing cell metabolic activity) [25] was performed by preparing 96-well plates and then the L 929 cells are seeded in each well. Then, the seeded cells are treated with different concentrations of synthesized nanoparticles such as 50, 100, 150, 200, and 250 µg mL^−1^. Then, the plates, are incubated for 24 h at 37 °C. Excess media was discarded and fresh media was added, then, 10 µL of tetrazolium dye was added and incubated for 4 h. After incubation, a color change from yellow to purple was observed. After incubation color change was observed from yellow to purple color and replaced with DMSO of 200 µL, OD was then taken [26]. 

#### 2.7.6. Cell Cytotoxicity Assay

The L 929 cell lines are seeded in 6-well plates and then treated to reach a confluent growth. Each cell area was treated with quercetin and different concentration of silver nanoparticles such as 50, 100, 150, 200, and 250 µg mL^−1^ and incubated for 24 h at 37 °C in a carbon dioxide incubator. Later, the supernatant of 50 µL was incubated in a carbon dioxide incubator and each well was treated with 2 mL of tris EDTA-NADH buffer and incubated for 30 min at 37 °C, then they were treated with fresh sodium pyruvate and the OD values were taken every 15 s for 3 min [26].

## 3. Results and Discussion

### 3.1. Isolated Strains

Nearly twenty samples were obtained from the laboratories and hospitals of Madurai (India), which were swabbed from the skin lesions. These samples were aseptically packed and brought to the college laboratory in time for further analysis. Among the isolates, the significant pathogens were identified as Gram-positive (4 isolates; 20%) and Gram-negative (16 isolates; 80%) as the result of gram staining and biochemical tests. 

### 3.2. AS-AgNPs Synthesis

A solution of silver nitrate (0.1 mM) was added to the 10 mL of *A. sessilis* leaf extract. After incubation, the reduction of the silver salt to silver shows a color change from golden yellow to bronze, visually confirming the synthesis of AgNPs.

### 3.3. Antibacterial Activity

**Figure 1:**
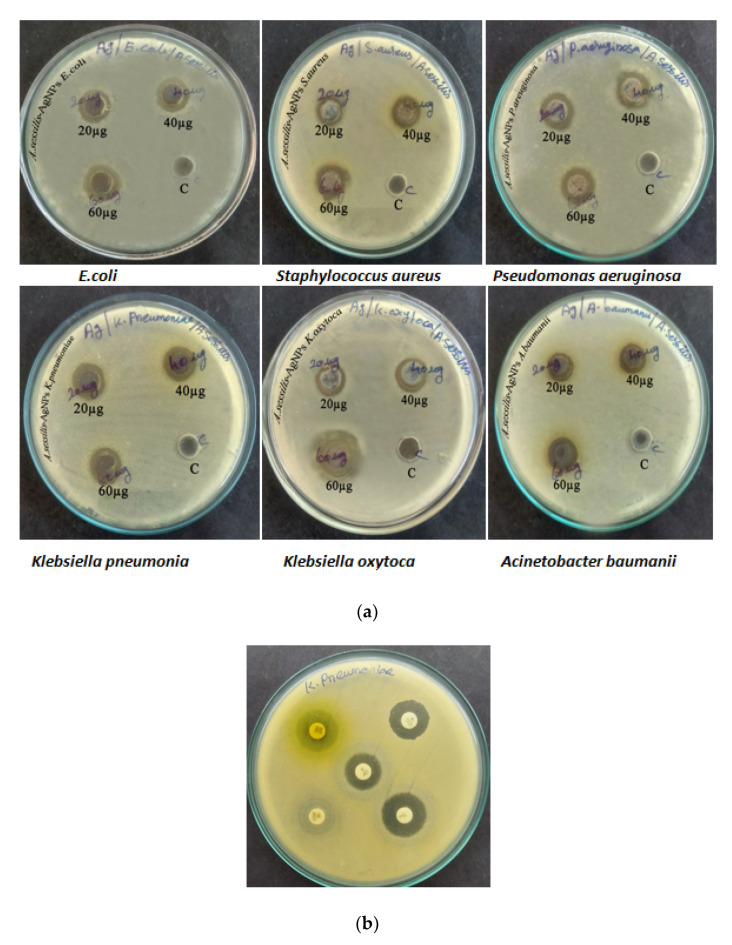
(**a**) Antimicrobial sensitivity of synthesized AS-AgNPs against the bacterial isolates. (**b**) Antimicrobial sensitivity of control against the bacterial isolate.

At this minimal inhibitory concentration, Gram-negative bacteria such as *K. pneumoniae* shows a 12 mm elevated zone of inhibition at a concentration of 60 µg/mL, indicating that is more susceptible than Gram-positive organisms such as *Staphylococcus aureus* which shows less inhibitory action concentration (11 mm at 60 µg/mL) against AS-AgNP (Table 1, Figure 1a). Clarithromycin (CLR15) antibiotic has kept as a positive control (Figure 1b). 

### 3.4. Fabric Sensitivity Test

**Figure 2:**
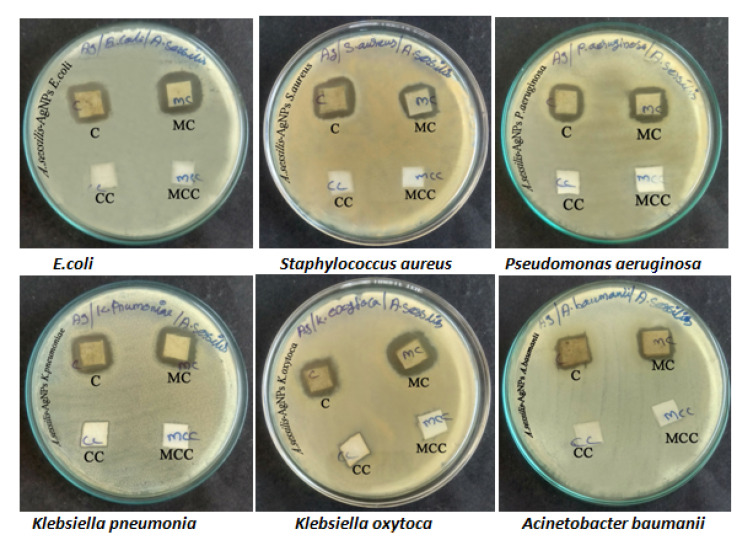
Antimicrobial synthesized AS-AgNPs embedded fabrics against all the bacterial isolates.

In Muller–Hinton agar (MHA), plate mixed cotton fabric soaked AS-AgNPs show more sensitivity against *K. pneumoniae* 12 mm, but in the case of Gram-positive organisms, *S. aureus* shows less sensitivity of 10 mm against the fabrics. Between these two fabrics, mixed cotton fabrics are more predominant. The results were inferred in Table 2 and Figure 2.

### 3.5. Conformational Study of AS-AgNPs Coated on Fabrics

#### 3.5.1. UV-Visible Spectrum

**Figure 3:**
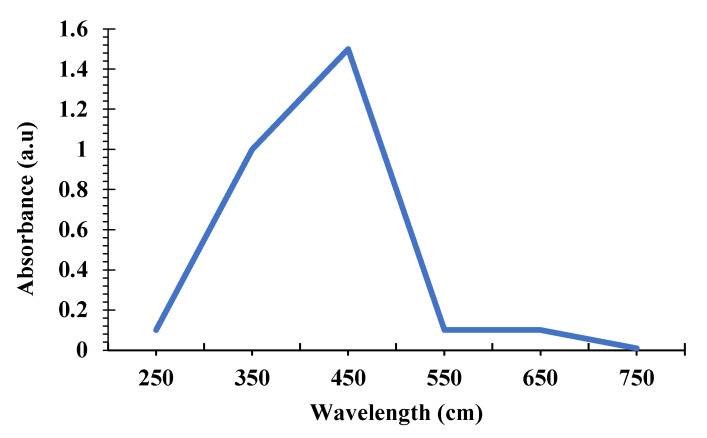
UV–Vis spectrum of synthesized AS-AgNPs.

The spectral analysis shows the presence of silver nanoparticles (Ag NPs) at a strong absorbance of 450 nm after 1 h of incubation. The slow rise of the peak starts from 350 nm and reaches up to 450 nm is evidence of the surface plasmon resonance peak (SPR) of silver. The intensity of the absorbance corresponding to its wavelength is depicted in Figure 3.

#### 3.5.2. FTIR (Fourier Transform Infrared Spectroscopy) 

**Figure 4:**
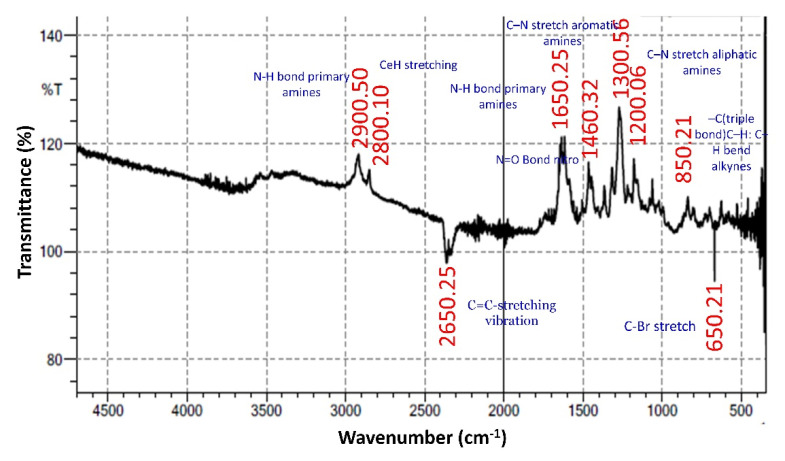
Spectrum of FT-IR analysis of synthesized AS-AgNPs.

FTIR analysis shows the presence of important chemical groups in the silver nanoparticles, where the AgNP would bind to the functional groups through bridges, the IR spectrum exhibits strong peaks at 550 cm^−1^, 15,000 cm^−1^ (probably 1500 cm^−1^) and 4000 cm^−1^” (When analyzing Figure 4, no signal is observed at 500 cm^−1^, the second signal described is at 1550 cm^−1^ and the third is centered at 3600–3700 cm^−1^) (Figure 4).

In particular the C=O stretching of carboxylic acid (1700–1730 cm^−1^) and ketone/aldehyde (1715–1725 cm^−1^). Similarly, the signals associated with alkenes are not clearly defined (1610–1690 cm^−1^ and 3010–3100 cm^−1^), nor are those O-H of acid groups (2400–3400 cm^−1^) or phenolic groups (3300–3650 cm^−1^) was compared with *Cydonia oblonga* leaf extract which similar phenolic groups in the range of (3301–3650 cm^−1^). The synthesized AS-AgNPs exposed the subsistence of carboxylic, alkene groups, and carbonyl groups. This ultimately evinced the accessibility of plentiful chemical components embedded in the synthesized AS-AgNPs.

#### 3.5.3. *SEM (Scanning Electron Microscope) with Energy Dispersive X-ray Analysis (EDAX)*

**Figure 5:**
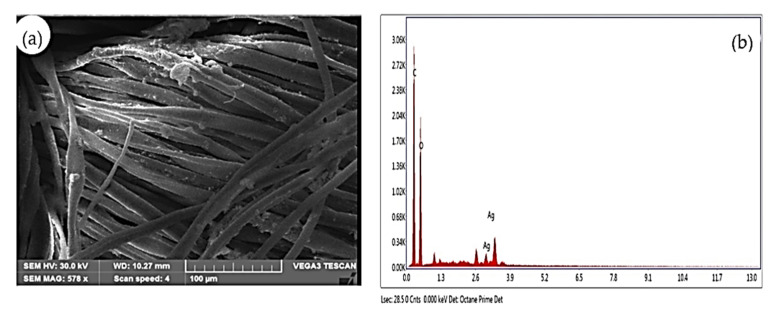
SEM (**a**) and EDX analysis (**b**) of AS-AgNPs coated cotton fabrics”, incorporate axis; axis X: “Energy (KeV)” and axis Y “Intensity (u.a.)”. The SEM analysis of synthesized AS-AgNPs embedded fabrics has exhibited the attachment of AS-AgNPs in the exterior of fabrics.

The SEM analysis was performed at a magnification of 578X. The size of the Ag NPs synthesized from the aqueous leaf extract of *A. sessilis* mediated silver nanoparticle coating on the fabrics is in the range of 10.27 nm. Thus, the obtained, results show that capping of the bioactive compounds from the aqueous leaf extract of *A. sessilis* and the Ag NPs are embedded on the fabrics. A picture was obtained with a voltage of accelerating electrons at 30 KV and magnification of 578X is shown in Figure 5a. In EDAX (Figure 5b) a metal atom from the nanoparticles with a peak in the range of 2.6–3.9 KeV was evidenced [27].

#### 3.5.4. *High-Resolution Transmission Electron Microscopy (HR-TEM)*

**Figure 6:**
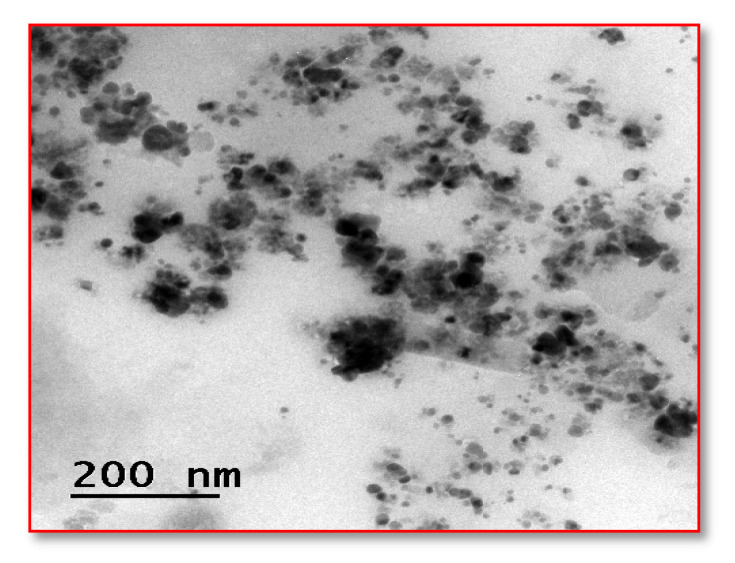
HR-TEM analysis of synthesized AS-AgNPs.

The HR-TEM investigation indicated that the synthesized AS-AgNPs are black-white crystalline, spherical structures and show a size distribution from 15–40 nm, and it was recorded by LCD camera (Figure 6).

#### 3.5.5. MTT Cell Viability Assay

**Figure 7:**
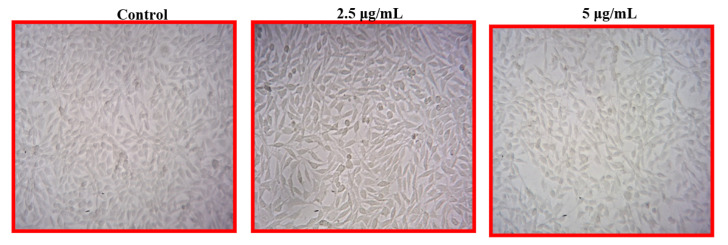
Morphological changes in control and As-AgNPs treated normal fibroblast L929 cells for 24 h. This figure shows photomicrographs (20X) where it is possible to see the morphology of sample 3 (Table 2) treated with L929 cells. Treatment of sample 3 did not show toxicity or cellular changes to the L929 cells, which is similar to the control.

The aqueous extract of *A. sessilis* mediated AgNPs was tested against L 929 cell lines and checked the viability through MTT assay. The AgNPs effectively show the inhibition of L929 cells at a minimal concentration of 5.0 µg/mL, and depending on the size of the synthesized AgNPs, the inhibitory action and the viability of the L929 cell line increases, and the positive slide and cell viability are depicted in Figure 7.

#### 3.5.6. Cell Proliferation Assay

**Figure 8:**
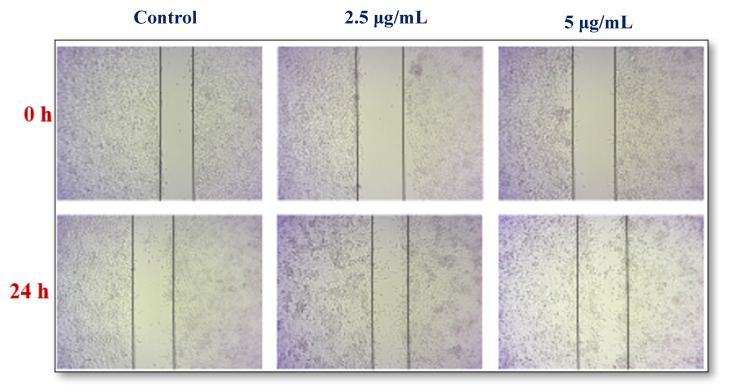
Effect of cell migration of As-ANPs treated against L929 cell lines.

The cytotoxic effect of the silver nanoparticles synthesized from aqueous leaf extract of *A. sessilis* treated with L929 cell lines and an estimation of the toxic effect of the silver nanoparticles are depicted in Figure 8. Cell migration of L929 cell lines treated with As-AgNPs shows no cytotoxicity after 24 h. The silver nanoparticles morphology and inhibitory action against L929 cell lines show induced cell senescence in a concentration-dependent manner. 

The preparation of AgNPs with the help of *A.* *sessilis* plant extract was evaluated for its wound healing potential. The leaf extract of *A. sessilis* after mixing with a silver nitrate solution leads to a reduction of silver ions by showing the color change from straw to bronze color within a few hours. This fact is indicative of the formation of silver nanoparticles [28]. The process of oxidation and the reduction are responsible for the color change, and therefore can be used as an indicator in the NPs synthesis. In the antimicrobial activity, the Gram-negative organism *K. pneumoniae* shows an inhibitory action of 12 mm at 60 µg mL against As-AgNPs. In the case of the fabric sensitivity test, the fabric coated with As-AgNPs shows an inhibitory action of 12 mm against *K. pneumoniae* than Gram-positive organisms. The UV-vis spectral analysis determines the surface plasmon resonance raised which indicates the formation of silver is started from 350 nm and obtained a sharp peak at 450 nm. The FTIR spectrum exhibited strong peaks at 550 cm^−1^, 1500 cm^−1^, and 3000 cm^−1^, which correspond to the presence of carboxylic, alkene groups, and carbonyl groups, respectively [29]. The reduction and stabilization of nanoparticles are due to the presence of phenolic compounds in the *A. sessilis* leaf extract and it was detected by phytochemical analysis. SEM analysis was performed at a magnification of 578X and depict the size range of nanoparticles at 10.22 nm. In EDAX, the presence of the elemental silver in the given extract of *A. sessilis* extract sample has evidenced a peak in the range of 2.6–3.9 KeV and it is also a confirmatory study of AgNPs. The size and morphology of the synthesized AgNPs are provided by HR-TEM showing agglomerated dispersed spherical shaped nanoparticles in the size range of 15–40 nm. Reducing the percentage of cell viability by the concentration of AgNPs, which has the capacity against L929 cell lines. Minimal concentration of AgNPs decreases the viability of the cell. A response curve was observed by the elevated concentration of AgNPs at 2.5 µgmL^−1^ with a decrease in the cell viability [30,31]. The concentration of AgNPs was treated in the range 2.5−5 µg/mL^−1^. In cell proliferation assay the dose concentration of AgNPs is between 80–120 µgmL.

## 4. Conclusions

In the present study, the biosynthesized silver nanoparticles play an important role in biomedical applications. The obtained silver nanoparticles from *A. sessilis* exhibit an active antibacterial potential related to the traditional drug. The cell lines assay exposes the silver nanoparticles clinical therapies like in vivo wound healing assay. This work also proves *A. sessilis* mediated silver nanoparticles also act as regenerative medicine. This work acknowledges that the metal microparticles along with *Alternanthera sessilis* enhance and pave the way for scavenging free radicals because it is toxic-free and eco-friendly, and acts as a vehicle to treat the biofilm on the ulcers. 

## Figures and Tables

**Table 1:** Antimicrobial sensitivity of synthesized AS-AgNPs and antibiotic against the bacterial isolates.

S. No	Name of the Organism	Zone Diameter (20 µg/mL)	Zone Diameter (40 µg/mL)	Zone Diameter (60 µg/mL)	Zone Diameter (Clarithromycin CLR 15 µg)
1.	*Escherichia coli*	8 mm	10 mm	12 mm	19 mm
2.	*Staphylococcus aureus*	6 mm	9 mm	11 mm	18 mm
3.	*Pseudomonas aeruginosa*	4 mm	6 mm	9 mm	28 mm
4.	*Klebsiella pneumoniae*	9 mm	10 mm	12 mm	17 mm
5.	*Klebsiella oxytoca*	10 mm	11 mm	13 mm	19 mm
6.	*Acinetobacter baumanii*	11 mm	12 mm	13 mm	6 mm

**Table 2:** Antimicrobial sensitivity test of fabrics (cotton and mixed cotton) against the bacterial isolates.

S.No.	Name of the Organism	Cotton Fabrics	Mixed Cotton Fabrics
1.	*E. coli*	09 mm	13 mm
2.	*Staphylococcus aureus*	10 mm	10 mm
3.	*Pseudomonas aeruginosa*	09 mm	11 mm
4.	*Klebsiella pneumoniae*	08 mm	12 mm
5.	*Klebsiella oxytoca*	08 mm	10 mm
6.	*Acinetobacter baumanii*	06 mm	08 mm

## Data Availability

Data sharing is not applicable for this article.
